# 3D QSAR studies, pharmacophore modeling, and virtual screening of diarylpyrazole–benzenesulfonamide derivatives as a template to obtain new inhibitors, using human carbonic anhydrase II as a model protein

**DOI:** 10.1080/14756366.2016.1241781

**Published:** 2017-03-19

**Authors:** Yeganeh Entezari Heravi, Hassan Sereshti, Ali Akbar Saboury, Jahan Ghasemi, Marzieh Amirmostofian, Claudiu T. Supuran

**Affiliations:** a Department of Chemistry, Faculty of Science, University of Tehran, Tehran, Iran;; b Institute of Biochemistry and Biophysics, University of Tehran, Tehran, Iran;; c Department of Medicinal Chemistry, School of Pharmacy, Zabol University of Medical Sciences, Zabol, Iran;; d Dipartimento Neurofarba, Universita degli Studi di Firenze, Sezione di Scienze Farmaceutiche, Florence, Sesto Fiorentino, Italy

**Keywords:** Carbonic anhydrase isoform II, molecular docking, pharmacophore, sulfonamide, virtual screening

## Abstract

A 3D-QSAR modeling was performed on a series of diarylpyrazole-benzenesulfonamide derivatives acting as inhibitors of the metalloenzyme carbonic anhydrase (CA, EC 4.2.1.1). The compounds were collected from two datasets with the same scaffold, and utilized as a template for a new pharmacophore model to screen the ZINC database of commercially available derivatives. The datasets were divided into training, test, and validation sets. As the first step, comparative molecular field analysis (CoMFA), CoMFA region focusing and comparative molecular similarity indices analysis (CoMSIA) in parallel with docking studies were applied to a set of 41 human (h) CA II inhibitors. The validity and the prediction capacity of the resulting models were evaluated by leave-one-out (LOO) cross-validation approach. The reliability of the model for the prediction of possibly new CA inhibitors was also tested.

## Introduction

Metalloproteins are an essential part of the human proteome and contain one or more metal-ion cofactors, being estimated that approximately 30% of all proteins in humans belong to this class^1^^,^^2^. Among all metalloproteins that have been identified, carbonic anhydrases (CAs, EC 4.2.1.1) play a significant role, due to their physiological functions in all living organisms. The CAs are a class of enzymes that catalyze the reversible hydration of CO_2_ to HCO_3_^−^ and a proton^3–6^. CAs can be classified into seven distinct classes, i.e. α-, β-, γ-, δ-, ξ-, η-, and θ-CAs^5^^,^^7–10^, which are distributed to various tissues and cells that are found in both prokaryotes and eukaryotes. Among these five classes, α-class is signified over other classes because it is found in all vertebrates. From the 16 isoforms that belong to the α-class, human carbonic anhydrase isoform II (hCA II) is the most physiologically abundant isoform, being found in red blood cells, secretory tissues, brain, gastro-intestinal tract, eyes, kidneys, etc.^11^^,^^12^.

The CA active site is located in a deep cavity of around 15 Å, at the bottom of which the catalytically crucial Zn^+2^ ion is bound by three histidine residues and a hydroxide ion/water molecule. The catalytic site is separated into hydrophilic and hydrophobic parts^4^^,^^5^^,^^13^. The main class of CA inhibitors (CAIs) is constituted by the primary sulfonamides which bind to the zinc ion as anions, replacing a water molecule nucleophile^13–17^.

Quantitative structure activity relationship (QSAR) is a technique that is used to identify and predict the protein–ligand interactions and to explore the relationships among molecular structures and biological activities. Accordingly, this technique plays an essential role in the process of drug design. In contrast to this technique, 3D-QSAR is a method that is utilized to calculate the highly specific interactions and states how far and with how much power a molecule can be connected to the active site of a protein or enzyme^18–21^.

In the present study, we have performed 3D-QSAR, using comparative molecular field analysis (CoMFA), CoMFA-region focusing (CoMFA-RF), and comparative molecular similarity indices analysis (CoMSIA) techniques on a series of 41 diarylpyrazole-benzenesulfonamide derivatives reported earlier^11^^,^^12^ to act as CAIs, in order to determine the influence of their fields on the inhibitory activity. The main goal of these techniques is to find an informative instruction between the 3D characteristics or structural changes of these compounds and their inhibitory potencies to understand the performance of these inhibitors on their target, hCA II. Using graphical contour maps derived from the three constructed models, features that are essential for the interaction of compounds with target protein were extracted and used to build a pharmacophore model in order to obtain a new scaffold for screening in a large chemical database to design new compounds as novel hCA II inhibitors with more inhibitory power. In addition, compounds that adapted with pharmacophore model were filtered by Lipinski’s rule of five and ADMET properties. Finally, the molecular docking protocol; GOLD was successfully utilized to orient these new compounds as hits in the active site of the protein and those with essential interaction with hCA II were selected based on their resemblance. Also these hits may act as novel leads for hCA II inhibitor design.

The compounds were chosen for these computational investigations due to their interesting scaffolds which have also been used for obtaining cyclooxygenase II (COX II) inhibitors^20^.

## Material and methods

### Data set

All diarylpyrazole-benzenesulfonamide inhibitors and their inhibitory potencies were collected from literature^11^^,^^12^. In order to obtain consistent numerical values; the *K_i_* (M) values were converted into the corresponding p*K_i_* (−log *K_i_*) whose activity ranges are from 6.1 to 8.4 log units provide a homogenous data for 3D-QSAR study. In order to carry out the analysis, a series of 41 diarylpyrazole-benzenesulfonamide derivatives were divided into a training set of 26, test set of 10 and validation set of five compounds (Table 1a–c). The test set was chosen on the basis of distribution of biological data as well as structural variation of inhibitors while the validation set was selected randomly without any prior assumptions.

**Table 1.** (a) Structures and experimental inhibitory potency (*K_i_*, nM) for compounds of the general structures Ι and II.

Structure I, compounds **1**–**12**		Structure II, compound 13	
Compound	*R*^1^	*K_i_* (nM)	p*K_i_*
**1**	Phenyl	420	6.376
**2**	Pyridyl	43	7.366
**3**	Tolyl	176	6.754
**4**	Bromophenyl	117	6.931
**5**	Cyanophenyl	122	6.913
**6**	4-Methoxyphenyl	136	6.866
**7**	3-Methoxyphenyl	237	6.625
**8**	2-Methoxyphenyl	153	6.815
**9**	1-Naphthalenyl	46	7.337
**10**	2-Naphthalenyl	9.6	8.017
**11**	6-Methoxy-2-naphthalenyl	264	6.578
**12**	Biphenyl	8.9	8.051
**13**	Phenyl	381	6.419

**Table 1.** (b) Structures and experimental inhibitory potency (*K_i_*, nM) for compounds of the general structures III, IV, and V.

Structure III, compound **17**		Structure IV, compounds **18**, **19**, and **20**		Structure V, compounds **14**, **15**, and **16**	
Compound	*R*^1^		*K_i_* (nM)		p*K_i_*
**14**	Phenyl		38		7.420
**15**	2-Naphthalenyl		36		7.443
**16**	6-Methoxy-2-naphthalenyl		31		7.508
**17**	2-Naphthalenyl		9.1		8.040
**18**	Phenyl		181		6.742
**19**	2-Naphthalenyl		490		6.309
**20**	6-Methoxy-2-naphthalenyl		782		6.106

**Table 1.** (c) Structures and experimental inhibitory potency (*K_i_*, nM) for compounds of the general structures VI, VII, and VIII.

Structure VI, compounds **21**–**28**		Structure VII, compounds **29**–**35**		Structure VIII, compounds **36**–**41**	
Compound	*R*^1^		*K_i_* (nM)		p*K_i_*
**21**	Phenyl		21		7.677
**22**	Tolyl		5.6		8.251
**23**	4-Methoxyphenyl		34		7.468
**24**	4-Bromophenyl		10.1		7.995
**25**	4-Chlorophenyl		4.5		8.346
**26**	4-Fluorophenyl		4		8.397
**27**	4-Nitrophenyl		361		6.442
**28**	Thienyl		9.5		8.022
**29**	Phenyl		240		6.619
**30**	Tolyl		321		6.493
**31**	4-Methoxyphenyl		325		6.488
**32**	4-Bromophenyl		560		6.251
**33**	4-Chlorophenyl		579		6.237
**34**	4-Fluorophenyl		312		6.505
**35**	4-Nitrophenyl		681		6.166
**36**	Phenyl		9.3		8.031
**37**	Tolyl		9.2		8.036
**38**	4-Methoxyphenyl		185		6.732
**39**	4-Bromophenyl		244		6.612
**40**	4-Chlorophenyl		178		6.749
**41**	4-Fluorophenyl		64		7.193

### CoMFA and CoMSIA analysis

In this study, predictive 3D-QSAR models CoMFA, CoMFA-RF (region focusing), and CoMSIA were performed on the entire dataset using SYBYL 7.3 molecular modeling software from Tripos, Inc. (St. Louis, MO). Before modeling with these two primary methods, the 3D structure of the inhibitors were sketched in SYBYL 7.3, then energy minimization were implemented using the Tripos force field with a distance-dependent dielectric and the Powell conjugate gradient algorithm (convergen cecriterion of 0.001 kcal/mol*A) and partial atomic charges were calculated using the Gasteiger–Hückel method. The aim of CoMFA is to derive a correlation between the biological activities of a set of molecules and their 3D structures. All molecules in a dataset have common substructures. Therefore, the most active molecule in a dataset was used as a main template and other molecules were superimposed on it. As a result, choosing an appropriate way to align the molecules on each other is very essential to make an efficient and robust model. Here, molecules were aligned on each other with field-fit method. The procedure of this alignment method is based on minimizing RMSD due to the six rigid body degrees of freedom and/or any user specified torsion angles^22^. The RMSD function is the sum of the steric and electrostatic energies averaged across all the lattice points between the molecule of interest and a template molecule. In CoMFA method, all aligned molecules were located at the center of grid box with a dimension of 2.0 Å using a sp^3^ hybridized carbon atom as a probe with a +1.0 charge to calculate steric and electrostatic interaction fields. The Coulomb and Lennard–Jones potential functions (Equations (1) and (2)) were used to estimate the steric and electrostatic interactions. As a default, column filtering was set to 2 kcal/mol in terms of reducing noise and attenuate signal to noise ratio but in this study it was set to 0.3 kcal/mol. The intended cutoff to compute both steric and electrostatic fields was set to 30 kcal/mol. It is noteworthy that the quality of the model is highly dependent on the direction of the aligned molecules in the 3D-gridbox; therefore, an all orientation search (AOS) was written in SYBYL programming language (SPL)^23^, which was used to optimize the field sampling by rotating the molecular aggregate systematically and selecting the orientation of aligned molecules in a grid box space in order to achieve the highest *q*^2^. Since the CoMFA model allocates the equal weight to data from each lattice point, we have utilized a complementary method, referred to as CoMFA region focusing (CoMFA-RF), which refines a model by improving the weight for those lattice points and enhance the contribution of these points which are most related to the model.

In a comparable manner, CoMSIA was employed to calculate five different fields of steric, electrostatic, hydrophobic, hydrogen bond acceptor, and hydrogen bond dono. It is important to note that the grid box in this method was analogous to the one in CoMFA model and the CoMSIA similarity indices descriptors were obtained with the same lattice box used in CoMFA. Therefore, to determine these similarities *A*_F,K_, the mutual distance between the probe atom (*W*_probe,k_) and each molecule atom is considered and calculated using Equation (3) for a molecule *j* with atoms *i* at a grid point *q* as follows^24^.

The standard settings of CoMSIA are explained as follows: a probe atom (*W*_probe,k_) with charge +1, radius 1 Å, hydrophobicity +1, hydrogen-bond donating +1, hydrogen-bond accepting +1, a column filtering of 2 kcal/mol (in this study 0.3 kcal/mol mentioned above), an attenuation factor of 0.3(*α*), and a grid spacing of 2 Å for the Gaussian type distance dependence were used to derive physico-chemical properties^25^. Following multiple attempts to assess all possible combinations of different fields, an optimal predictive CoMSIA model was acquired using five similarity indices descriptors namely; steric, electrostatic, hydrogen bond donor, and hydrogen bond acceptor and also hydrophobic fields, which were available within SYBYL. Furthermore, to establish a linear relationship between the calculated energy values in both CoMFA/CoMSIA methods and experimental values (p*K_i_*), partial least square (PLS) algorithm was used^26^:

(1) $$E_{c}=\;\sum_{i=1}^{n}\frac{q_{i}q_{j}}{D\;r_{\mathrm{ij}}}\;$$

(2) $$E_{\mathrm{vdW}}=\sum_{i=1}^{n}(A_{\mathrm{ij}}r_{\mathrm{ij}}^{-12}-\;C_{\mathrm{ij}\;}r_{\mathrm{ij}}^{-6})\;$$

(3) $$A_{F,K}^{q}\left(j\right)=\;-\sum W_{\mathrm{prob},k}W_{\mathrm{ik}}e^{\alpha r_{\mathrm{iq}}^{2}}$$

### Pharmacophore modeling

Pharmacophore modeling was carried out to generate common feature pharmacophore model with the catalyst/HipHop program in Discovery Studio 2.5 (Accelrys, San Diego, CA). It must be noted that using this protocol can generate pharmacophores that are common to a set of active ligands and optionally it can add excluded volumes to the pharmacophore model by entering a series of inactive compounds. In fact, the purpose of a pharmacophore model is to provide a set of steric and electrostatic features that are vital for an optimal interaction with specific biological target^27^. Prior to the generation of pharmacophore hypotheses, all 3D structures of some of the most active compounds from training set were sketched in SYBYL 7.3 (Tripos Associates, Inc., St Louis, MO), then energy minimized by the Powell method with a gradient of 0.001 and maximum iteration was adjusted to 3000. Subsequently, to obtain the best conformation of the stated compounds, they were exported to Discovery Studio 2.5 software (Tripos Associates, Inc., St Louis, MO) for docking analysis. Before docking analysis, CHARMm force field was applied on these compounds and then partial charges were calculated using Momany–Roneoption. At this moment, the compounds are ready for generating conformations using GOLD algorithm. The most stable conformation with the highest GOLD score fitness, and also the most appropriate interaction with the target protein (hCAII) was selected as bioactive conformer for each compound in the training set as four of the most active ones. Other parameters that were chosen for generating conformations including maximum pharmacophore: 10, maximum features: 10, minimum features: 4, conformation method: best, energy threshold: 5 kcal/mol, maximum conformations: 255.

### Molecular docking

Molecular docking is one of the most frequently used methods in drug design because of its ability to predict how small molecules interact with the appropriate target binding sites. Molecular docking was used to investigate the mode of interaction of target protein (hCAII) to its inhibitors to find the most stable configuration that is similar to the bioactive one. Herein, the crystal structure of human carbonic anhydrase II (hCAII) 3QYK was taken from RCSB protein databank (http://www.pdb.org). Since there is no report in PDB of this protein complex with any studied ligands within the dataset, in docking study, its original ligand was removed and those in our dataset were docked sequentially into the active site of hCAII. For the preparation of ligands prior to docking, their structures were built using the SKETCH module as implemented in SYBYL 7.3 (Tripos Inc., St. Louis, MO) running on a Red Hat Linux workstation 4.7 and their geometry was optimized using the MINIMIZE module. The minimization process uses the POWELL method with the TRIPOS force field to reach a final convergence of 0.001 kcal/mol. The genetic algorithm based docking program GOLD was used to dock inhibitors into the active pocket of hCA II. Actually, it uses an evolutionary algorithm that gives the full flexibility to the various ligand conformations and considers a relative flexibility to the protein. Also, this program optimizes the fitness score by using a genetic algorithm and gives the scoring function that is dimensionless^28^. The GOLD score function has been optimized for the prediction of ligand binding positions and takes into account factors such as H-bonding energy, van der Waals energy, metal interaction, and ligand torsion strain. However, the scale of the score indicates how good the pose is: the higher the score, the better the docking result. Docking simulation was then performed into hCAII (RCSB Protein Data Bank 3QYK) with the automated GOLD program which has been incorporated into Discovery Studio 2.5 (Accelrys Software Inc., San Diego, CA)^29^. In the next step, the 3D structure of the protein was imported to the Discovery Studio environment and subsequent measurements were carried out by applying CHARMm force field^30^, all water molecules were removed and hydrogen atoms were added, and then pH of the protein was adjusted to almost neutral, 7.4, using protein preparation protocol (Momany–Rone partial charge method). The later operation was followed by using the smart minimization algorithm of Discovery Studio 2.5 (Accelrys Software Inc., San Diego, CA), which performs 1000 steps of steepest descent with a RMS gradient tolerance of 3 and conjugate gradient minimization. The X-ray crystallographic data revealed that target protein has only one chain A with a length of 260 amino acid residuesand a bounded ligand namely IE2 (4-(7-methylpyrazolo [3',4':4,5] thiopyrano[2, 3-b] pyridin-1(4H)-yl)benzenesulfonamide and a water molecule. The catalytic active site of hCAII consists of two amino acids of Glu 106 and Thr 199 that play a crucial role in the interaction of inhibitors with the enzyme through hydrogen bond with the hydroxyl group of Thr199, which in turn is bridged with the carboxylate moiety of Glu 106. These interactions strengthen the nucleophilic property of zinc ion bound to water molecule and provide a favorable orientation of substrate (CO_2_) or inhibitors of this enzyme for nucleophilic attack^4^. Prior to removing the reference ligand, a 10 Å radius sphere was defined around the protein to illustrate the ligand binding site and active site residues surrounding the bounded ligand. Finally, to validate the docking procedure and determine how GOLD algorithm can predict the minimal energy pose, root-mean square distance (RMSD) was calculated between the bounded inhibitor and redocked ligand (IE2), which was 1.14 Å in this method. This value represents the validity of GOLD method to reproduce the known binding mode of inhibitors in the dataset.

### Virtual screening

The best pharmacophore model was used as a 3D query for searching in databases. The purpose of virtual screening is to prevent broad searches in a large chemical space and evaluating very large libraries of small molecules that are capable of becoming drug using computer programs. Generally, the databases contain compounds that are commonly accessible and relative inexpensive. In this study, four of the most active compounds from the initial dataset were chosen and docked with the target protein (hCAII) using GOLD algorithm. Then, by performing this operation we could obtain 10 conformers from each compound. They also ranked from the highest to the lowest score fitness. Eventually, the best conformer with highest score and appropriate interactions with enzyme (hCAII) was chosen as a bioactive conformation for further analysis.

The selected conformers were imported to the Discovery Studio software (Accelrys Software Inc., San Diego, CA) and the pharmacophore model was built following a structural alignment. This can lead to the emergence of common pharmacophoric features. This process can also be performed with a single active compound in the dataset, but the former makes the pharmacophore model more valuable and credible. At last, we uploaded the most active compound (**26**) to the ZINCPharmer site, which contains 176 million conformers of 18.3 million compounds^31^ and built a pharmacophoric features, then the coordinates of the main pharmacophore model (obtained from the most active aligned compounds) applied to compound **26** and use it for virtual screening of a subset of ZINC database. The primary filters were performed in this software as follows: molecular weight (*M*_W_ ≤300), total number of rotatable bond (≤10) and maximum hits per conformation equal to 1 were applied as the first filter, which yielded a subset of 309 hit compounds. Of these compounds, those containing unsubstituted sulfonamides were chosen. Accordingly, 62 compounds were selected and passed through a second filtration Lipinski’s rule of 5^32^ to ensure that the selected compounds possess the basic properties of a drug compound. Using this filtration confirms drug-like properties of these hits. At this stage, all 62 compounds could pass through this filter successfully. Here are some terms of governing this rule that compounds must have these conditions to be capable as a drug for virtual pharmacokinetic testing. (i) *M*_W_ of less than 500 Da; (ii) an octanol–water partition coefficient log *P* not greater than 5; and (iii) hydrogen bond donor, hydrogen bond acceptor sites (N and O atoms) no more than 5 and 10, respectively.

Finally, to investigate the interactions between compounds and target protein (hCAII), all 62 filtered hits were imported to Discovery Studio 2.5 software (Accelrys Software Inc., San Diego, CA) package in order to conduct molecular docking analysis for further narrow down the retrieved hits using GOLD docking protocol.

### ADME studies

There is no guarantee that the compound with the best interactions with target protein is not necessarily a good medicine. Many factors must be considered in order for a molecule to become a drug. After the passage of molecules from filters discussed in the previous section, now it is time to check the compounds by virtual pharmacokinetic testing before synthesize them for biological tests. To achieve this goal, ADME studies were conducted. ADME is the acronym of four major topics in pharmacokinetics: absorption, distribution, metabolism, and excretion/elimination of a drug. It also includes a number of tests to describe the path of a New Chemical Entity (NCE) within the animal or human body, and it is evident that poor pharmacokinetics in the human body can indicate a primary reason for drugs failure^33^. Of the relationships between the chemical structures and physiological properties, we can calculate some pharmacokinetic characteristics that gain useful information about the function of the compounds in the body which are supposed to be as inhibitors. In the following discussion we mentioned some pharmacokinetic characteristics as important descriptors for each compounds that would be a drug such as polar surface area (PSA), blood brain barrier (logBB)^33^^,^^34^, log *K*_hsa_ for serum protein binding, skin-permeability coefficient (log *K*_p_), the octanol–water partition coefficient (log *p*), and other distribution descriptors like apparent Caco-2 or MDCK permeability. All these descriptors are essential for any compounds to traverse of cell membrane and reach the target tissue such as binding to receptors. For instance, polar surface area (PSA)^35^ is commonly used in medicinal chemistry and it is used for the optimization of a drug’s ability to permeate cells. In fact, molecules with a PSA greater than 140 Å^2^ tend to be poor at permeating cell membranes and those with PSA less than 90 Å^2^ are quite well to penetrate the blood–brain barrier to act on receptors in the central nervous system. Another important parameter is the octanol–water partition coefficient (log *p*) that shows the drug hydrophobicity and expresses that compounds with higher hydrophobicity have an increased metabolism and low absorption that may inadvertently be connected to other lipophilic molecules, hence increase the potential toxicity. ADME descriptors were computed by using QikProp v. 3.2 programs (Schrodinger, Portland, OR, 2009) and were checked with the related standard ranges. Experimental results that were used in developing QikProp (Schrodinger, Portland, OR) are for more than 710 compounds including about 500 drugs and related heterocycles^36^. Here, all 62 compounds were evaluated in terms of ADME parameters and all were within the allowable range. Subsequently, the outputs were imported in MATLAB as their descriptors and illustrate the chemical space of only 49 compounds that have better ADME properties using score plot. After that those are structurally similar and dissimilar to each other (10 compounds) were chosen (five similar and five dissimilar). This similarity/dissimilarly can be traced back to the minimum and maximum Euclidean distance in the chemical space of the compounds. Accordingly, the structure–activity relationship study (SAR) examined on those compounds that in addition to their structural similarity, they have the ability to synthesis. To illustrate this assertion, Figure SF1 (Supplementary data), represents the score plot of these compounds to show their chemical space. As can be seen in Figure SF1, red and green circles represent compounds that are structurally similar and dissimilar, respectively. More detailed description is given in Virtual screening section.

### PLS analysis and validations

PLS regression was carried out on the dataset to build 3D-QSAR models using standard implementation in the SYBYL 7.3 package (Schrodinger, Portland, OR). The goal of this method is to find a correlation between descriptors based on CoMFA and CoMSIA models and inhibitory potency of compounds, as well as construct a linear regression model by projecting predicted and observed variables into a new space. In this study, molecular interaction fields which were determined in CoMFA and CoMSIA analyses and p*K_i_* values were used as interpretive and dependent variables in PLS regression analysis, respectively. Leave-one-out (LOO) cross-validation method was employed as an internal validation in order to obtain the optimal number of components (latent variables) with a minimum standard error of estimate and the highest cross-validated correlation coefficient *q*^2^^37^ that was calculated by Equation (4). It is necessary to mention that by using the same number of components, non-cross-validation method was performed to calculate conventional *r*^2^. External validation (r_pred_^2^) was used for estimating the accuracy and reliability of the model as well as predictability of the model was estimated by validation set. Both internal and external validations were performed on training set and test set, respectively. Finally, in order to improve the signal to noise ratio in 3D-QSAR, based on CoMFA and CoMSIA models, column filtering was set to an optimum value according to the model built:

(4) $$q^{2}=1-\frac{\sum_{i=1}^{\mathrm{training}}(y_{i}-y_{i}^{\wedge }){}^{2}}{\sum_{i=1}^{\mathrm{training}}(y_{i}-y_{i}^{-}){}^{2}}$$

## Results and discussion

### CoMFA and CoMSIA analyses

In this study, statistical parameters of the CoMFA, CoMFA-RF, and CoMSIA model were built to evaluate the reliability of constructed 3D-QSAR model, and the results were reported in Table 2. At first, PLS data analysis for both CoMFA and CoMFA-RF was carried out. According to the results, the *q*^2^ value for CoMFA and CoMFA-RF was equivalent to 0.703 and 0.742 with three components, respectively, which is indicative of the reliability and robustness of these models. Then, statistical results were compared with each other and showed that the CoMFA-RF is better than common CoMFA. Therefore, at first, CoMFA model was performed on the entire dataset without any processing, and then to achieve the highest *q*^2^, we used the AOS method that was previously described. Based on the procedure of this method, after obtaining the highest *q*^2^ value, the dataset was divided into three parts: training, test, and validation set. Then, the CoMFA model was performed on the training set and statistical parameters were calculated. It should be noted that the statistical results of CoMFA-RF and CoMSIA models were obtained according to the same manner that was expressed for CoMFA model. Figure SF2 (Supplementary data) depicts the actual p*K_i_* against predicted p*K_i_* values for the compounds in the training, test, and evaluation sets based on CoMFA, CoMFA-RF, and COMSIA models. Other statistical parameters were as follows: r_ncv_^2^ = 0.856 and 0.862, r_pred_^2^ = 0.891 and 0.742, *F* value (Fischer ratio) of 43.584 and 45.959, SEE (low standard error of estimation) of 0.312 and 0.305 with a column filtering of 0.3 kcal/mol for both CoMFA and CoMFA-RF, respectively.

**Table 2.** Summary of the results obtained from the CoMFA, CoMFA-RF, and CoMSIA models.

Component	CoMFA	CoMFA-RF	CoMSIA
*q*^2^	0.703	0.742	0.555
R_ncv_^2^	0.856	0.862	0.818
RMSEC	0.458	0.28	0.383
n	3	3	3
RMSEP	0.226	0.319	0.308
*F* value	43.584	45.959	32.927
R_pred_^2^ (test set)	0.891	0.742	0.743
R_pred_^2^ (validation set)	0.790	0.720	0.922
(R^2^ - R_0_^2^)/R^2^	0.08	0.00	0.07
*K*	1.001	1.02	1.009
Fraction			
Steric	0.500		0.065
Electrostatic	0.500		0.249
Hydrophobic			0.191
H-bond donor			0.391
H-bond acceptor			0.103

q^2^: cross-validated correlation coefficient after the leave-one-out procedure;

R_ncv_^2^: non-cross-validated correlation coefficient; RMSEC: root-mean-square-error for training set; *n*: optimum number of components;

RMSEP: root-mean-square-error for test set; R_pred_^2^: predictive correlation coefficient; R_0_^2^: correlation coefficient for regression through origin for predicted versus observed activities; *K*: slope of regression lines through the origin.

Another model that was performed in this article is CoMSIA, and the number of features that were used was a combination of five fields. In addition to the steric and electrostatic features in CoMFA model, hydrophobic, hydrogen bond donor and hydrogen bond acceptor descriptors were employed to build CoMSIA model. The statistical results obtained from a combination of these five fields with the same amount of components in CoMFA method are r_pred_^2^ = 0.743, *F* value of 32.927 and SEE = 0.350 with a column filtering of 0.3 kcal/mol. The contribution of each field illustrates the importance of them on building a model. In CoMFA model, the contribution proportion of both steric and electrostatic features were similar to each other, also in CoMSIA, the results suggest that the combination of these five fields has a significant impact on constructed model; therefore, from the data provided in Table 2, it can be asserted that the contribution of hydrogen bond donor feature is more than any other features used in CoMSIA model. In addition, Table 2 demonstrated additional statistical characteristics in terms of estimating the predictive power of 3D-QSAR model. These parameters which have been proposed by Golbraikh and Tropsha are as follows:

$$\frac{\left(R^{2}-\;R_{0}^{2}\right)}{R^{2}}0.1\;\mathrm{or}\;\frac{R^{2}-R_{0}^{'2}}{R^{2}}0.1$$

$$0.85\leq K\leq 1.15\;\mathrm{or}\;0.85\leq K^{'}\leq 1.15$$

where *R^2^* is the predictive correlation coefficient for the predicted p*K_i_* versus the experimental observed values for test set compounds; R_0_^2^ and R_0_^'2^ are the coefficients of determination for regression lines through the origin between predicted versus observed activities and observed versus predicted activities, respectively. Moreover, K and K^'^ are the slopes of the regression lines when forcing the intercept through origin for predicted versus observed activities and vice versa. The alignment of all compounds in the dataset was done in SYBYL program (Certara USA, Inc., Princeton, NJ) using field fit alignment method. In addition, the values of experimental activities (p*K_i_*) versus predicted activities and their residuals (Exp p*K_i_* – Pred p*K_i_*) are listed in Table 3.

**Table 3.** Experimental and predicted inhibitory activities (p*K_i_*) with residual values for the training and test set compounds.

		CoMFA		CoMFA-RF		CoMSIA	
Compound		p*K_i_*	Pred.	Res.	Pred.	Res.	Pred.	Res.
1*‡	6.376751	6.774	−0.39725	7.23	−0.85325	6.972	−0.59525
**2**¶	7.366532	6.953	0.413532	7.36	0.006532	7.026	0.340532
**3**¶§	6.754487	6.893	−0.13851	7.041	−0.28651	6.998	−0.24351
4*†‡	6.931814	6.846	0.085814	6.991	−0.05919	6.729	0.202814
**5**	6.91364	6.835	0.07864	6.78	0.13	7.015	−0.10136
**6**	6.866461	6.773	0.093461	6.83	0.03	7.066	−0.19954
**7**¶§	6.625252	6.495	0.130252	6.991	−0.36575	7.02	−0.39475
8*†‡	6.815309	6.955	−0.13969	7.105	−0.28969	6.992	−0.17669
9*†‡	7.337242	7.369	−0.03176	7.505	−0.16776	7.008	0.329242
10*	8.017729	8.016	0.001729	7.107	0.910729	7.068	0.949729
**11**	6.578396	7.406	−0.8276	6.81	−0.24	6.672	−0.0936
**12**‡	8.05061	8.088	−0.03739	7.9	0.15	7.924	0.12661
**13**¶§	6.419075	6.376	0.043075	7.188	−0.76892	7.049	−0.62993
**14**	7.420216	7.452	−0.03178	7.53	−0.11	7.315	0.105216
**15**	7.443697	7.342	0.101697	7.46	−0.02	7.446	−0.0023
**16**	7.508638	7.388	0.120638	7.58	−0.08	7.474	0.034638
**17**	8.040959	8.101	−0.06004	8.11	−0.07	7.902	0.138959
18*†‡	6.742321	6.836	−0.09368	7.477	−0.73468	7.256	−0.51368
**19**	6.309804	6.346	−0.0362	6.17	0.13	6.132	0.177804
**20**	6.106793	6.152	−0.04521	6.25	−0.15	6.138	−0.03121
**21**¶§	7.677781	8.262	−0.58422	8.148	−0.47022	8.245	−0.56722
**22**	8.251812	8.095	0.156812	8.17	0.08	8.289	−0.03719
**23**	7.468521	8.564	−1.09548	7.95	−0.49	8.028	−0.55948
24*†‡	7.995679	7.565	0.430679	8.054	−0.05832	8.111	−0.11532
**25**	8.346787	8.248	0.098787	8.06	0.28	8.095	0.251787
**26**	8.39794	8.218	0.17994	8.04	0.35	8.03	0.36794
27*	6.442493	6.795	−0.35251	8	−1.56	8.062	−1.61951
**28**	8.022276	8.174	−0.15172	8.3	−0.28	8.259	−0.23672
29*†‡	6.619789	6.683	−0.06321	6.484	0.135789	6.479	0.140789
30*†‡	6.493495	6.534	−0.04051	6.299	0.194495	6.519	−0.0255
**31**	6.488117	6.617	−0.12888	6.24	0.24	6.262	0.226117
**32**	6.251812	6.292	−0.04019	6.23	0.02	6.306	−0.05419
**33**	6.237321	6.333	−0.09568	6.24	−0.01	6.32	−0.08268
**34**	6.505845	6.759	−0.25315	6.29	0.21	6.269	0.236845
**35**	6.166853	5.786	0.380853	6.35	−0.19	6.292	−0.12515
**36**	8.031517	8.070	−0.03848	7.43	0.6	7.37	0.661517
**37**	8.036212	7.953	0.083212	7.42	0.61	7.419	0.617212
**38**	6.732828	7.915	−1.18217	6.86	−0.13	7.142	−0.40917
**39**	6.61261	7.451	−0.83839	7.06	−0.45	7.193	−0.58039
**40**	6.74958	7.464	−0.71442	7.23	−0.49	7.202	−0.45242
**41**	7.19382	7.951	−0.75718	7.35	−0.16	7.169	0.02482

*Test set for CoMFA.

†Test set for CoMFA-RF.

‡Test set for CoMSIA.

¶Validation set for CoMFA/CoMFA-RF.

§Validation set for CoMSIA.

### CoMFA contour maps analysis

**Figure 1.**
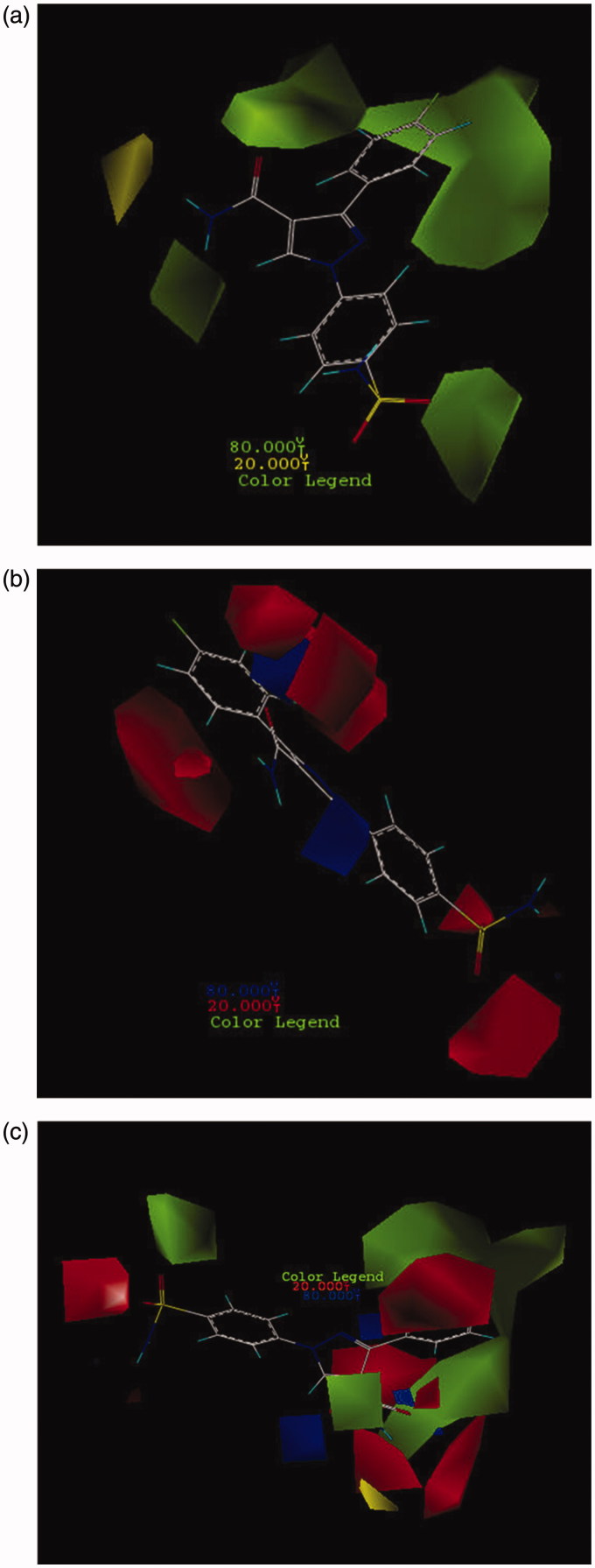
CoMFA contour maps based on compound **26**: (a) steric, (b) electrostatic, and (c) consolidated steric and electrostatic.

In CoMFA- and CoMSIA-based 3D-QSAR models, contour maps were used as an informative tool to visualize the interaction between inhibitors and target protein (hCAII). The colors for these graphical contour maps express suitable interaction areas in inhibitors and hCAII. In CoMFA model, both steric and electrostatic fields were identified by the 3D contour maps with specific colors, green, and yellow contours illustrate the favorable and unfavorable regions in terms of spatial for steric feature, respectively. Additionally, red and blue contours represent areas that are desirable in terms of electro negativity and electro positivity that in most cases the contribution of these contours are 80% and 20%. All contours that were achieved in both models along with their interpretation are depicted in the following section. Here in this work, steric (green and yellow) and electrostatic (blue and red) fields for CoMFA model were shown as 3D contours on the most active compound in a dataset (compound no. **26**) as a template and depicted by consolidated and separate contours in Figure 1(a–c).

It is clear that the change in substituents in both steric and electrostatic fields along with maintaining pharmacophore groups can alter the biological activities of compounds. According to the results that can be seen in (Figure 1a), green contours indicate that bulky groups can lead to increase the biological activity especially in the area of phenyl substituted, amine from amide group and one of sulfonyl groups. Here are some terms of governing this rule as well as, by placing small groups in the regions of a molecule (NH_2_ of –CONH_2_) that are exactly positioned on the opposite site of bulky areas can lead to increase the activity of compounds. As shown in Figure SF3, a comparison between compounds that are more active than acetazolamide (AZA) indicates that all these compounds have bulky groups surrounding phenyl substituent and NH from amide group. Table 4 shows that these compounds have common substructures. The common parts are displayed in red. As illustrated, pyrazole 5-sided ring was not used as a pharmacophore and its existence was just only a linker that was confirmed in molecular docking study. Therefore, modifying its orientation does not alter the biological activity and also binding of compounds to the active site of the protein. All these factors have caused the compounds in the active site of hCA II rotate in such a direction that their essential functional groups were exactly located in regions of protein, which are favorable in terms of steric and electrostatic. Another part of all molecules which were observed in all four substructure categories (shown in Table 4) is benzenesulfonamide functional group. Keeping this group leads to increase biological activity of entire molecules in the dataset. The blue part in these four categories does not have an important role in binding to the active site. In addition, this part does not have an impact on the inhibitory potency. Areas that are shown in green are important and so due to pyrazole rotation, molecules were oriented in the green contour maps properly and leading to increase their activity. Compounds **36** and **37** which contain hydrazine groups have high inhibitory potency due to the expansion of substitution in an appropriate direction of NH_2_ from -CONH_2_ group. Also, Figure SF4 demonstrates the aligned compounds that are less active than acetazolamide (AZA). Comparison between the most and the least active compounds than AZA showed that lacking the desired substitution of these compounds in the relevant part of green contours lead to reduce their activity. Hence, the low activity of these compounds can be attributed to their electrostatic characteristics. Figure 1(b) shows electrostatically favor and disfavor (blue and red) contour maps on the most active compound (**26**) in the dataset. As shown, red contours around the substituent on the pyrazole and sulfonamide rings indicate that by placing electron acceptor groups in this area, the activity of compounds increases. Furthermore, in compound **26**, the presence of 4-fluoro-phenyl has significantly increased the activity of this compound. This confirms that there are amino acids in the active site of hCAII that possess electron donor property; therefore, they can interact with their complementary functional groups of a compound with an electron acceptor property. Since the sulfonamide groups are electron acceptor, they can bond with amino acids His119 and Thr199 correctly. The blue areas in small sizes that are surrounding the pyrazole-5-sided ring demonstrated that by placement of much restricted electron donor groups in these areas the compound’s activity can be increased. Figure SF5 shows that all active compounds than AZA have the same pattern of substitution in these areas and the presence of the SO_2_ group as a vital pharmacophore in a red area showed that this group has an electron acceptor characteristic.

**Table 4.** Structural form of the more active compounds than acetazolamide (AZA).

Investigations of CoMFA contour maps indicate that both steric and electrostatic fields have a similar impact on the activity of molecules. The results obtained in Table 2 confirmed this claim as the proportion of both steric and electrostatic features are equal to 0.5. In other words, the presence of electron donating substituent might have reduced the activity of a molecule; in addition, by locating it in appropriate bulky areas the activity increases similar to compound **10**.

### CoMSIA contour maps analysis

**Figure 2.**
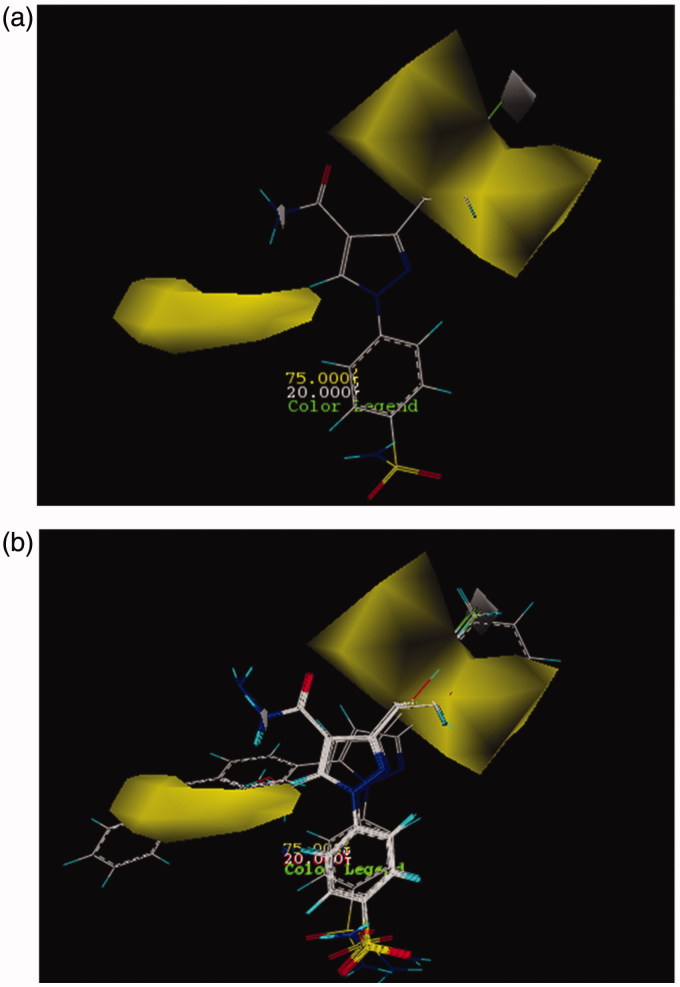
CoMSIA contour maps for hydrophobic and hydrophilic features: (a) for compound **26** and (b) for more active compounds than AZA.

**Figure 3.**
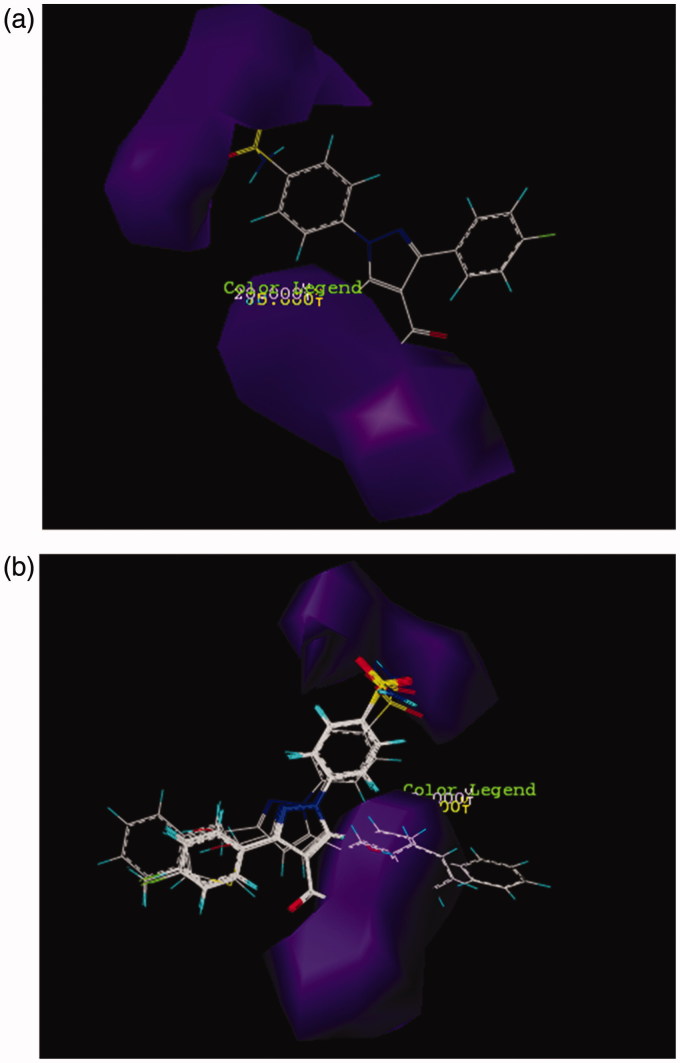
CoMSIA contour maps for the absence and the presence of hydrogen bond donor features based on (a) compound **26** and (b) more active compound than AZA.

**Figure 4.**
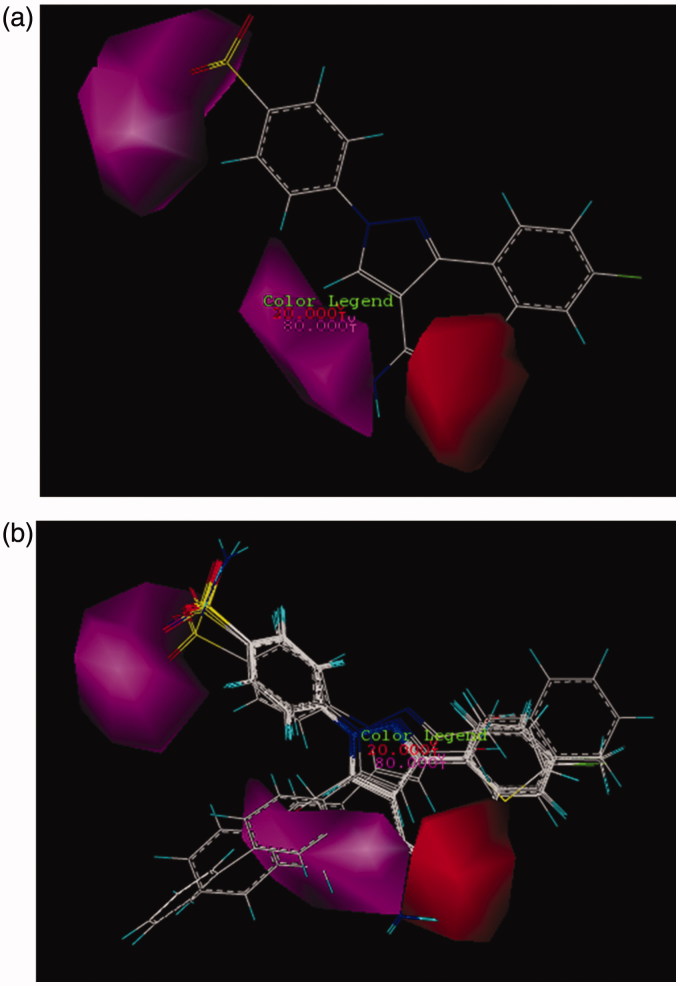
CoMSIA contour maps for the absence and the presence of hydrogen bond acceptor features based on (a) compound **26** and (b) more active compound than AZA.

In CoMSIA model, the distribution of all five fields named steric, electrostatic, hydrophobe, hydrogen bond donor, and hydrogen bond acceptor were shown and interpreted. Then the effect of these fields on the activity of molecules was described. Figure SF6 demonstrates the steric features on the most active compound **26** and represented that areas around phenyl substituent and pyrazole ring are favorable in terms of steric field. Moreover, its activity increased using low volume groups surrounding the area of amine from the amide group that was depicted as a yellow contour. The results of the steric contours in CoMSIA model are in accordance with the results obtained in the CoMFA model. As shown in Figure SF7, by placing the electron accepting groups in the area of pyrazole ring, sulfonamide group, and phenyl substituent, the activity of molecule 26 has increased. Moreover, the presence of blue contours (electron donating groups) in trace amounts showed that substituting a very small electron donating groups in this area can raise the activity of a molecule. Here in this case, the result of the study of electrostatic contours in CoMSIA model confirms the information attained from electrostatic contours in CoMFA model. Figure 2(a) shows the CoMSIA model for hydrophobic and hydrophilic groups which were illustrated as yellow and white 3D contours, respectively. The bulky yellow contour in the area of substituted phenyl indicated that by replacing huge lipophilic groups in this area, the activity of molecules can be increased. Also all active compounds than AZA (Figure 2b) have hydrophobic groups in phenyl substituted part and because of this reason have higher activity than AZA. Likewise, the activity of both compounds **17** and **12** were increased due to possess hydrophobic substituents surrounding the pyrazole ring. In CoMSIA model, there are violet and cyan contours that show the absence and the presence of hydrogen bond donor, respectively. In Figure 3(a), the violet contours around the nitrogen that belongs to an amide group, pyrazole ring, and also sulfonamide groups imply that as how hydrogen bond donor decreases in this area, the activity of a compound **26** will increase. Checking more active compounds than AZA (Figure 3b) indicated that because of lacking of hydrogen bond donor groups in mentioned regions, the activity of such compounds has increased. Furthermore, in CoMSIA model, pink and red contours demonstrate the presence and the absence of hydrogen bond accepting groups, respectively, and all are shown on compound **26**. As it turns out in Figure 4(a), contours of hydrogen bond accepting groups are located in the area of NH_2_ from benzosulfonamide, also in one side of pyrazole ring which was illustrated with pink contour. In contrast, red contours which are related to the groups that do not have the ability of accepting hydrogen bonds are located on the carbonyl group (C = O). Likewise, the more active compounds than AZA also follow this pattern (Figure 4b). In this way, the sulfonamide group just accepts hydrogen bond. Also if there are hydrogen bond acceptors on one side of pyrazole ring, then the activity of compounds can be increased. Results from Figures 3(a) and 4(a) can deduce that amino group does not play a role in accepting and donating hydrogen bond hence does not have any effect on the inhibitory potency of a compound 26. As can be seen from its docking study (Figure SF8, Supplementary data), the hydrogen bonds between compound **26** and its surrounding amino acids verify the results of CoMFA and CoMSIA studies. Thus, nitrogen atom in the benzosulfonamide group is capable to form hydrogen bond with hydrogen atom in the hydroxyl group of Thr199. Also hydrogen atom of the sulfonamide amine group can react with oxygen atom of the hydroxyl group of Glu 106. Both two sulfonamide groups (S=O) have an ability of accepting hydrogen bond with hydrogen atom of the amino group in Thr 199 and NH of His 119.

### Pharmacophore modeling analysis

By using ligand-based pharmacophore modeling, compound **26** was imported to ZINCPharmer site as the most active compound with a *K_i_* value of 4 nM in the dataset. The way it works is that common chemical properties among all under study compounds are obtained and a primary pharmacophore model was created. Initial features that were included in this qualitative pharmacophore model are hydrogen bond donor and hydrogen bond acceptor characteristics, hydrophobicity, and aromaticity. The resulting model which is gained from ZINCPharmer site is shown in Figure SF9 (Supplementary data). Aromaticity properties are related to both two phenyl and pyrazolrings; hydrophobicity based on fluorine, two phenyl rings, and pyrazolic group; hydrogen bond donor and hydrogen bond acceptor properties that are, respectively, related to hydrogens of the sulfonamide amino group, hydrogen’s of the –CONH_2_ group, oxygens of the SO_2_ group, nitrogen from pyrazol ring, and oxygen of the –CONH_2_ group exist around the active compound **26**. In order to enhance the accuracy of the pharmacophore model, the most four active compounds (**12**, **22**, **25**, and **26**) in the dataset were selected and superimposed to each other. Finally, the more accurate model was obtained with the following features and is depicted in Figure SF10 (Supplementary data). As can be concluded from this model, the obtained pharmacophoric features are of paramount importance and are essential for the inhibitory activity of the compounds. It is obvious that these properties are partly preserved to some extent because of this reason; they are active toward their target. Moreover, for designing of carbonic anhydrase inhibitors, the pharmacophoric portions of compounds should be maintained that they can have inhibitory activities.

### Virtual screening

Pharmacophore model obtained in the previous section was used for virtual screening. After the election of convenient features of this model that can play a vital role in the interaction between inhibitors and their target protein (hCAII), this model was used as a new scaffold to select and determine hit compounds from ZINC chemical database. The initial number of hits that were consistent with the pharmacophore model was 319. In order to restrict the number of hit compounds, Lipinski’s rule of 5 was performed on all 319 hits and its outputs were 216 compounds. According to the laws governing this filtration, there should be no more than five hydrogen bond donors and also no more than 10 hydrogen bond acceptors, molecular weight less than 500 Daltons, and an octanol–water partition coefficient (log *P*) not greater than 5. Then, visual inspection was carried out on their structures purposefully and the results showed that 62 compounds of the obtained 216 hits have sulfonamide groups which is the main part of all these compounds supposed to interact with their target (hCAII). Actually, according to investigation and previous researches on this series of compounds, molecules that have sulfonamide groups, especially those with free and unsubstituted sulfonamide, have high inhibitory activities toward hCAII. In the next stage, the compounds were ready for doing virtual pharmacokinetic testing. In fact, for obtaining the pharmacokinetic parameters of retrieved compounds, ADMET properties like absorption, distribution, metabolism, excretion, and toxicity were evaluated and all these 62 hits were able to pass this filter successfully. Some of the properties that are the results of this examination are: molecular weight (MW), (log *K*p) for skin-permeability, apparent Caco-2, log *P* for octanol/water, apparent MDCK permeability, and log BB for blood brain barrier were computed. It is obvious that all these compounds that could pass the filter were in acceptable ranges that are defined for 95% of known drug-like compounds. The results for the three final compounds with their structures and GOLD fitness score values are summarized in Tables 5 and 6. Finally, in order to choose the final compounds as mentioned in ADME Studies section, a series of the most important parameters obtained in ADME properties (seven parameters are shown with an asterisk in Table 6) were used as descriptors. Although all these 62 compounds could pass through the ADME filter, but only 49 of them were selected as imported objects to MATLAB because of their better properties. Therefore, we made a matrix with a dimension of 62 × 12 for analyzing the principal component analysis (PCA). Then by using data obtained from score plot (Figure SF1), the compounds lineup in the space of descriptors turned out. Therefore, three compounds were chosen that two of them (ZINC IDs: 36639942 and 36639437) are similar to each other from the physiochemical properties and one (ZINC ID: 13913968) is dissimilar with the other two hits. As it is clear from the data presented in Table 6, the molecular weight value for the final three hits indicates that these compounds are in the approved range, also this value (*M*_W_) for compound **26** as the most active compound and AZA as standard compound were 222.2 and 360.3, respectively. According to the results obtained in this study, compound **26** together with two other hits ZINC36639942 and ZINC36639437 was powerful as oral absorption model. From this point of view, AZA has an average power and a hit (ZINC13913968) showed low ability in this case. At the end, they were also computed for drug likeness study (OSIRIS property explorer 2012). Actually risk assessment of toxicity was evaluated for 3 hits, compound **26**, and AZA. From the results obtained in Table 7, four parameters of mutagenecity (MUT), tumorigenecity (TUM), irritating effects (IRR), and reproductive effects (REP) were examined. The results indicate that AZA has a risk of tumorigenecity as well as it can also have an adverse effect on reproduction. In this sense, compound 26 has no toxicity risk parameters. Both two hit compounds ZINC36639942 and ZINC13913968 exactly follow the same pattern as compound **26**, while another one (ZINC36639437) has high risks of irritating effects. Based on data from toxicity parameters as well as examine interactions of these hit compounds with their target (hCAII) which was explained in next section, it can be hoped that hit compound (ZINC36639942) can inhibit the hCAII as an appropriate drug for an investigation into production and development of drug.

**Table 5.** Structures and GOLD fitness score values for the hit compounds.

No. of hits	ZINC ID	Structures	GOLD fitness score
1	ZINC36639942		64.78
2	ZINC36639437		61.19
3	ZINC13913968		65.12

**Table 6.** Prediction of ADME properties of hits using Qikprop.

Descriptors	ZINC36639942	ZINC36639437	ZINC13913968	Compound no. 26	AZA	Stand. range*
*M*_W_	427.492	431.524	438.518	360.362	222.236	130.0–725.0
†Skin-permeability coefficient (log Kp)	−3.427	−3.5	−4.2	−4.712	−5.927	−8.0 to −1.0, K_p_ in cm/h
Jm, max transdermal transport rate	0.002	0.001	0.000	0.000	0.000	Micrograms/cm^2-h
Qual. Model for human oral absorption	High	High	Low	High	Medium	>80% is high
†Apparent Caco-2 permeability (nm/s)	114	94	32	45	35	<25 poor, >500 great
†% human oral absorption in GI (± 20%)	76	76	70	62	44	<25% is poor
†log *S* (aqueous solubility)	−5.025	−5.3	−6.4	−4.173	−1.412	−6.5 to 0.5
†log *P* for octanol/water	2.105	2.3	2.65	0.898	−1.850	−2.0 to 6.5
No. of primary metabolites	4	6	2	1	1	1.0–8.0
Apparent MDCK permeability (nm/s)	69	54	14	31	20	<25 poor, >500 great
†log *K*_hsa_ (serum protein binding)	−0.085	−0.05	0.29	−0.257	−0.974	−1.5 to 1.5
†log BB for brain/blood	−1.935	−2.2	−2.8	−1.949	−1.741	−3.0 to 1.2

*For 95% of known drugs, based on Qikprop v.3.2 (Schrodinger, Portland, OR, 2009) software results.

†Parameters imported in MATLAB as main descriptors.

**Table 7.** Prediction of drug likeness parameters and assessment of risk factors by OSIRIS Property Explorer.

Risk factors of toxicity					Drug-likeness parameters				
Name	MUT*	TUM†	IRR‡	REP¶	MW§	CLPǁ	S#	DL**	PSA††
ZINC36639942	None	None	None	None	427.504	3.944	−6.108	5.504	151.910
ZINC36639437	None	None	High	none	431.536	3.411	−5.073	4.965	148
ZINC13913968	None	None	None	None	438.531	2.564	−5.436	5.181	151.09
Compound 26	None	None	None	none	360.368	0.530	−3.431	5.030	129.45
AZA‡‡	None	High	None	High	222.249	−0.535	−1.636	3.503	151.66

*Mutagenicity.

†Tumorigenicity.

‡Irritating effects.

¶Reproductive effects.

§Molecular weight.

ǁcLog *P*.

#Solubility.

**Drug-likeness.

††Polar surface area.

‡‡Acetazolamide (standard material).

### Molecular docking results

Molecular docking analysis was used to study the interactions between hit compounds derived from virtual screening in the previous section and the target enzyme (hCAII). For this purpose, GOLD program was used and optimizes the fitness score by using a genetic algorithm. To validate the reliability of docking study, root-mean-square distance (RMSD) value was calculated between the cocrystal (bounded) and redocked ligand which was found to be 1.14 Å. This value shows a reliability of the GOLD method to reproduce the experimentally binding mode of hits as inhibitors. Results from the docking study suggest that all three hit compounds contain interactions with enzyme which are vital for their inhibition mechanism. For instance, hit compound (ZINC36639942) has the ability to form 6 hydrogen bonds (Figure SF11, Supplementary data) as follows: hydrogen of NH can interact with oxygen atoms (OE1 and OG1) from Glu106 and Thr199, respectively. Also both two oxygen atoms from the SO_2_ group form hydrogen bonds with NH of Thr199 and nitrogen (ND1) of His 119. Thiazol ring sulfur and amide group oxygen are oriented toward hydrogen of Gln92 and Asn67 to form hydrogen bonds, respectively. Figures SF12 and SF13 (Supplementary data), each one represents the hydrogen interactions between two remaining hit compounds (ZINC36639437 and ZINC13913968) and hCAII. Actually ZINC36639437 has established five hydrogen bonds with some vital amino acid in the active pocket of hCAII in which two of them belong to NH from the SO_2_NH– group with oxygen atoms (OE1 and OG1) of Glu106 and Thr 199, respectively. Also as it is clear in Figure SF12, an oxygen atom of sulfonamide group interacts with NH of His 119 and other oxygen interacts with NH of The 199. As well as NH of the amide group has the ability to form hydrogen bond with oxygen atom of Pro201. Figure SF13 shows the hydrogen bond interactions of ZINC13913968. As it is obvious, the ability to make hydrogen bonds between this hit and hCAII is not like the previous two hits, thus it seems that its inhibitory potency is less than other two hits. Also, as is clear from the molecular docking results, this hit can form four hydrogen bonds (two hydrogen bonds of pyrrole NH with oxygen atom of Pro201 and OG1 from Thr200, an oxygen atom of SO_2_ with NH of Thr199 and an oxygen atom of thiazol ring with hydrogen atom of Trp5; HE1) at the best condition that does not contain any hydrogen bond with Glu106 as an important amino acid in the active site of hCAII; therefore, it can also be attributed the low inhibitory power of this hit to this reason, but in other two hits hydrogen bond interactions with these two key amino acids (Glu106 and Thr199) are preserved.

Assessment resulting from the interaction of molecule **26** (the most active compound) with hCAII implies the existence of five hydrogen interactions with key amino acids in the active site of the enzyme that most of these interactions is that of forming a hydrogen bond from sulfonyl group’s oxygen with Thr199 and His119, as well as NH of the sulfonamide group (SO_2_NH–) which is bonded to Glu106 and Thr199 (Figure SF8). Also MOLCAD surface structure was calculated for the most active compound (**26**) into the active site of hCAII to illustrate the electrostatic potential surface. In fact, blue and red colors in the MOLCAD surface map showed the lower and the upper limit of the electrostatic potential. These colors represent the greatest amount of electronegative and electropositive potential, respectively. As can be seen in Figure SF14 (Supplementary data), it is obvious that the existence of the sulfonyl group (SO_2_) in compound **26** near to the red zone indicating that the electron accepting group at this site of a molecule has led to increase the inhibitory potency. The results of docking studies confirm the fact that there are some amino acids in this region such as Thr199 and His119 with hydrogen bond donor property that is complementary to the sulfonyl group, thus because of this issue, the MOLCAD surface of this area is shown in red. Also CoMFA and CoMSIA contour maps were verified this matter. Comparisons of the MOLCAD surface structure between the most (**26**) and the least active compounds (**20**) in the dataset showed that why the molecule **20** has lower activity than **26**. As shown in Figure SF15 (Supplementary data), this molecule is completely rotated in the active site of hCAII so that the distance between sulfonamide group nitrogen and Zn ^+2^ ion is more than 9 Å, this shows that there is no effective bond between compound **20** and its target; therefore, it has become a much weaker inhibitor in the dataset. Since sulfonyl is an electron accepting group, thus the supplementary section of it must have electron donor characteristics but as shown in its MOLCAD potential surface, this part is demonstrated with blue color that indicates the presence of electronegative groups. Actually this issue is an anomalously that should be. Another reason for the low inhibition of compound **20** can be because of the location of the sulfonamide group in a meta-position relative to the pyrazole ring. Experimental results investigated from the literature^11^^,^^12^ demonstrate that all three compounds (**18**, **19**, and **20**) with substructures that are depicted in Table 1(b) exhibited relatively low inhibitory potency.

## Conclusion

In the present paper, we reported a series of 41 diarylpyrazole benzosulfunamide derivatives as hCAII inhibitors with the aid of pharmacophore modeling and virtual screening to build a new scaffold for designing new hCAII inhibitors with high inhibitory power relative to their origins. In this regard, 3D-QSAR based on CoMFA/CoMFA-RF and CoMSIA was used as the powerful tools for making mathematical models in order to predict the inhibitory potency of the compounds, which are supposed to be a new generation of hCAII inhibitors. Eventually, interpretation of obtained contour maps from these models together with molecular docking analysis provides key structural features and also required interactions, which are efficient for inhibitory activity of these inhibitors. From the statistical parameters given in this study, we can conclude that these new compounds exhibit enhanced inhibitory activity and are suitable for further experimental analysis.

## Supplementary Material

IENZ_1241781_Supplementary_Material.pdf
