# Supplementary material for: 3D QSAR studies, pharmacophore modeling, and virtual screening of diarylpyrazole–benzenesulfonamide derivatives as a template to obtain new inhibitors, using human carbonic anhydrase II as a model protein
Source: J Enzyme Inhib Med Chem. 2017 Mar 19;32(1):688–700. doi: 10.1080/14756366.2016.1241781 (PMC6009914; doi:10.1080/14756366.2016.1241781)
Supplement: IENZ_1241781_Supplementary_Material.pdf [file IENZ_A_1241781_SM9777.pdf]

Supplementary data:

Figure SF1: Score plot of the retreived compounds from virtual screening.

Figure SF2: Plot of observed versus predicted activities for the training (filled red diamond), test (filled blue circle) and validation set (filled green triangle) molecules based on CoMFA, CoMFA-RF and CoMSIA models

Figure SF3: Steric CoMFA contour maps for more active compounds than AZA (acetazolamide).

Figure SF4: Steric CoMFA contour maps for the less active compounds than AZA.

Figure SF5: Electrostatic CoMFA contour maps for more active compounds than AZA.

Figure SF6: CoMSIA contour maps for steric fields based on compound 26.

Figure SF7:. CoMSIA contour maps for electrostatic fields based on compound 26.

Figure SF8: The docking result of the compound 26 as the most active compound in the dataset with hCAII by GOLD algorithm.

Figure SF9: The initial pharmacophore model of compound 26 made by ZINCPharmer site.

Figure SF10: The superposition of the most four active compounds (12, 22, 25 and 26) in the dataset for building qualitative pharmacophore model.

Figure SF11: The binding mode of the best conformer of the hit compound (ZINC36639942) with hCAII.

Figure SF12: The binding mode of the best conformer of the hit compound (ZINC36639437) with hCAII.

Figure SF13: The binding mode of the best conformer of the hit compound (ZINC13913968) with hCAII.

Figure SF14: The MOLCAD surface structure depicted with electrostatic potential of the hCAII active site using compound 26. The electrostatic potential color ramp ranges from blue (most electronegative potential values) to red (most electropositive potential values).

Figure SF15: The MOLCAD surface structure depicted with electrostatic potential of the hCAII active site using compound 20 as the least active compound in the dataset.

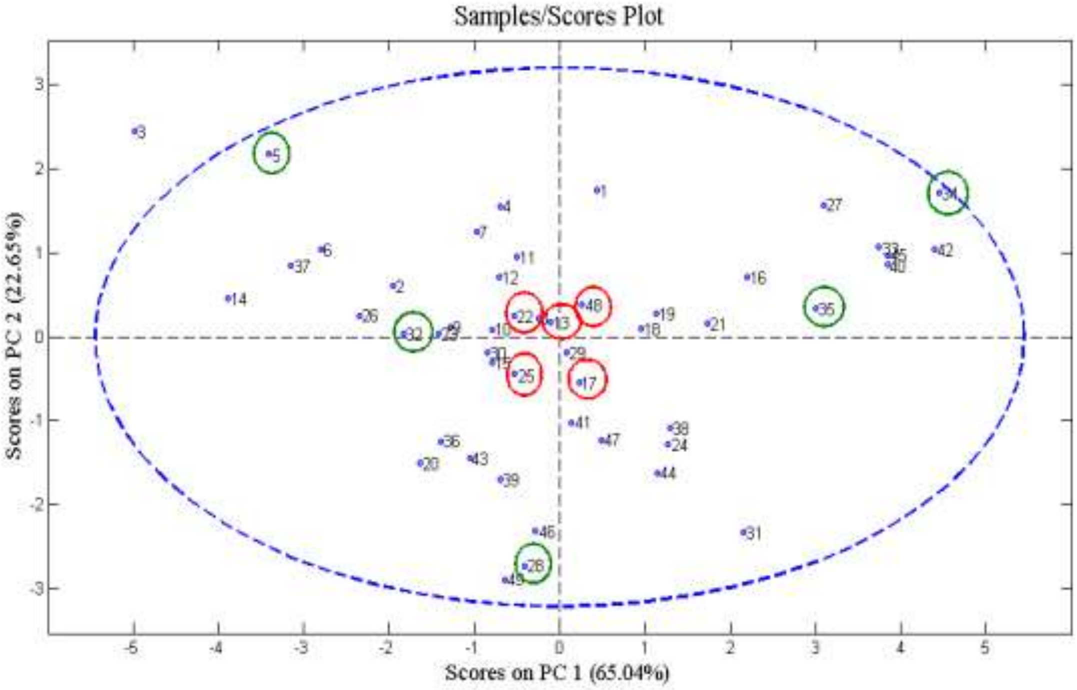
Samples/Scores Plot
Scores on PC 2 (22.65%)
Scores on PC 1 (65.04%)

Figure SF1

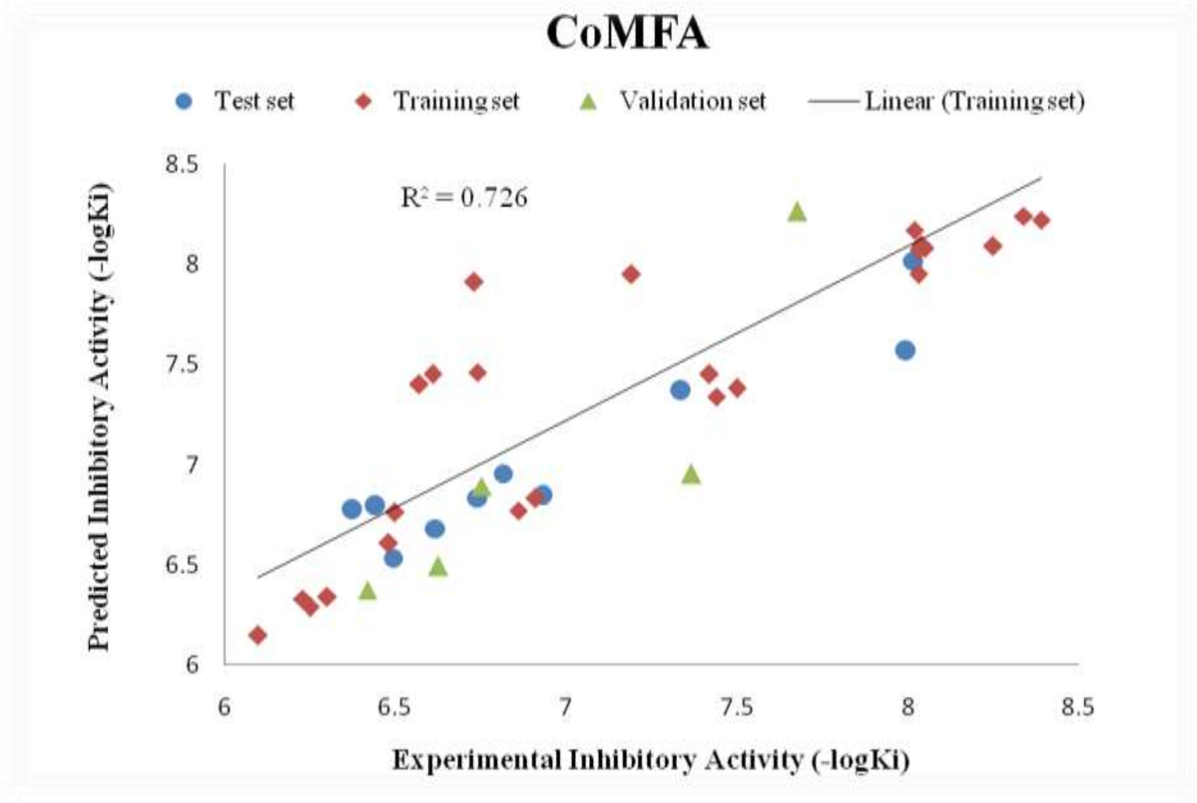
CoMFA
Test set
Training set
Validation set
Linear (Training set)
$R^2 = 0.726$
Predicted Inhibitory Activity (-logKi)
Experimental Inhibitory Activity (-logKi)

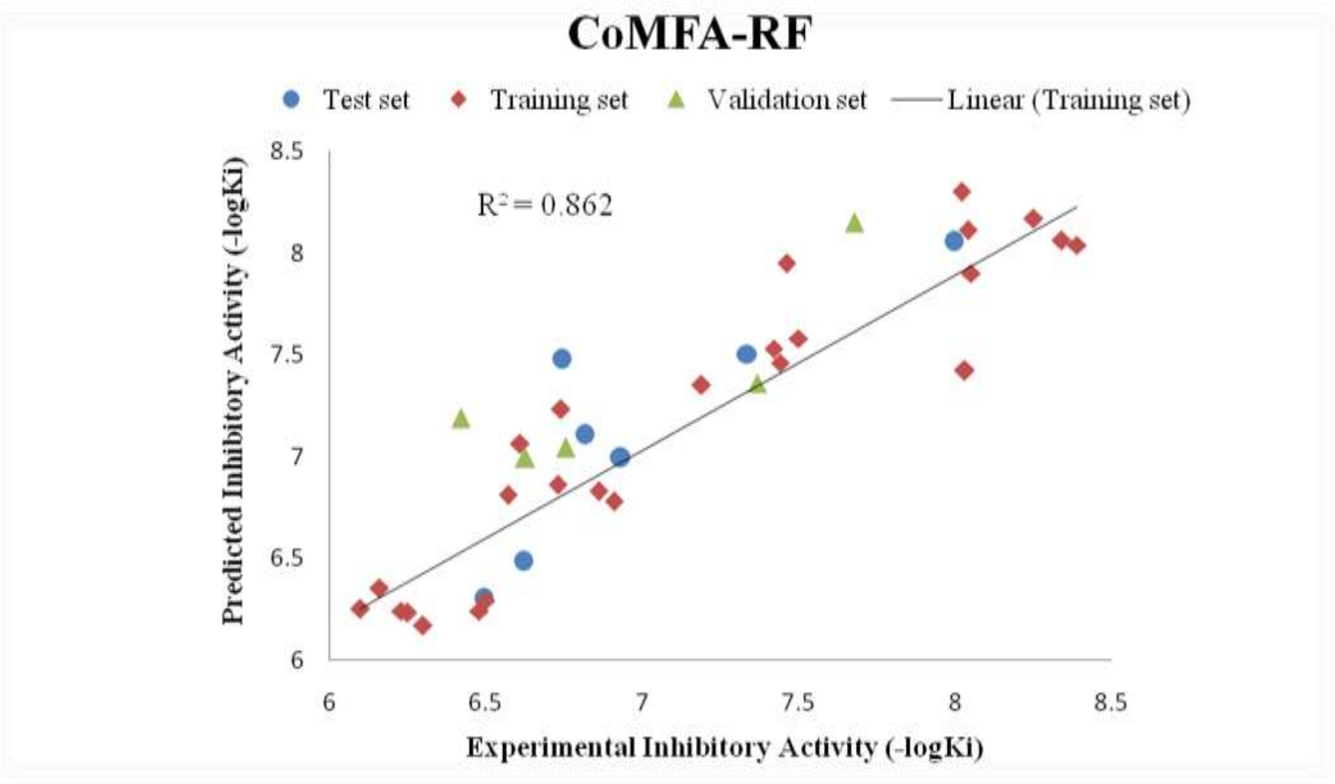
CoMFA-RF
Test set
Training set
Validation set
Linear (Training set)
R² = 0.862
Predicted Inhibitory Activity (-logKi)
Experimental Inhibitory Activity (-logKi)
6
6.5
7
7.5
8
8.5

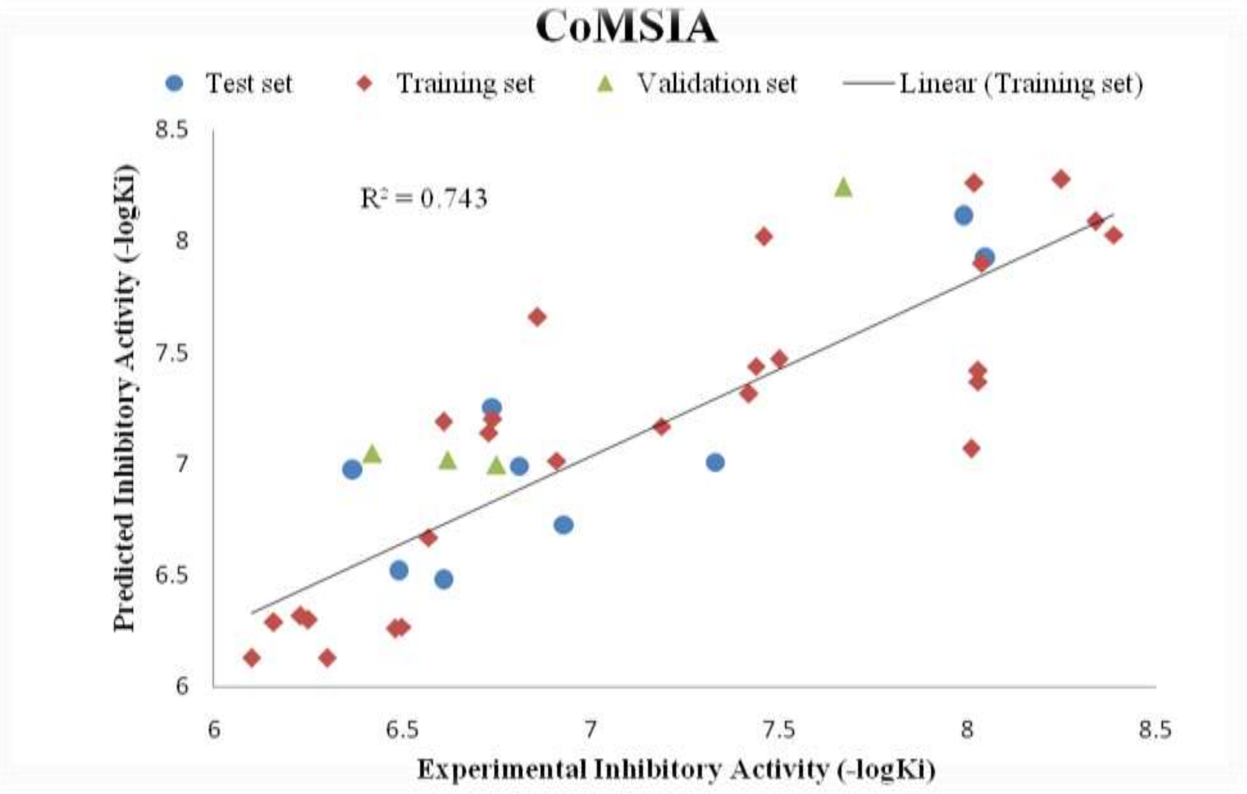
CoMSIA
Test set
Training set
Validation set
Linear (Training set)
R² = 0.743
Predicted Inhibitory Activity (-logKi)
Experimental Inhibitory Activity (-logKi)
6
6.5
7
7.5
8
8.5

Figure SF2

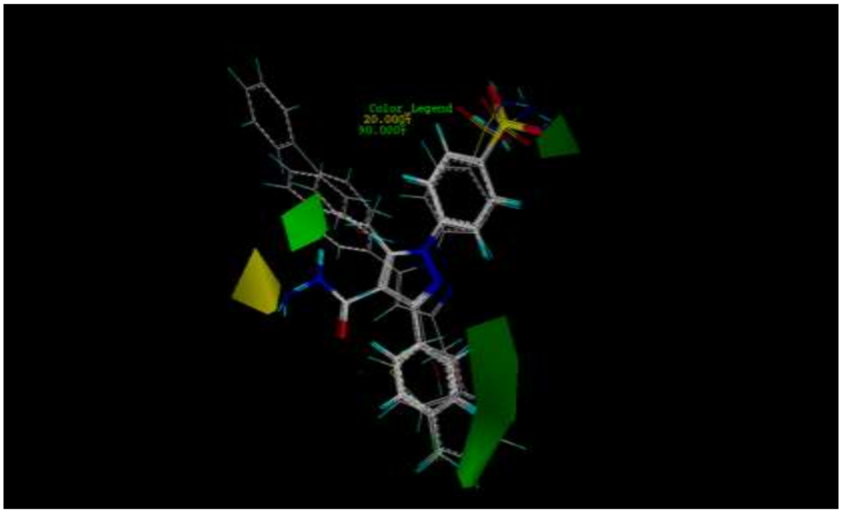

Figure SF3

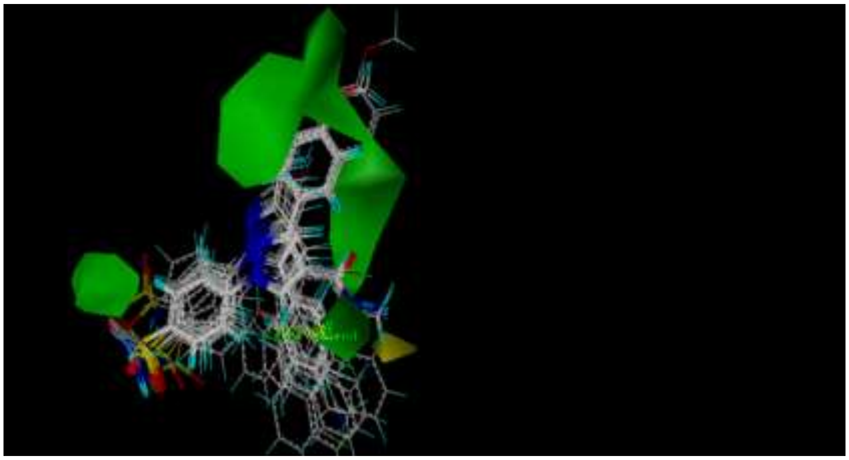

Figure SF4

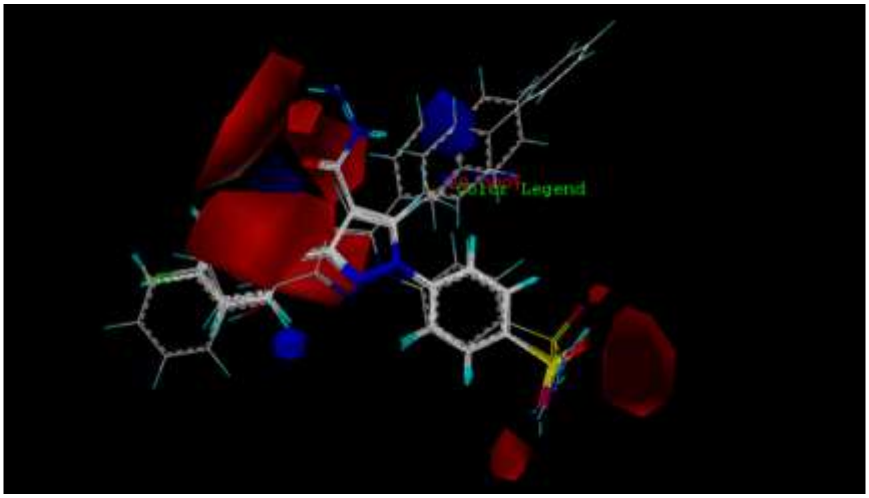

Figure SF5

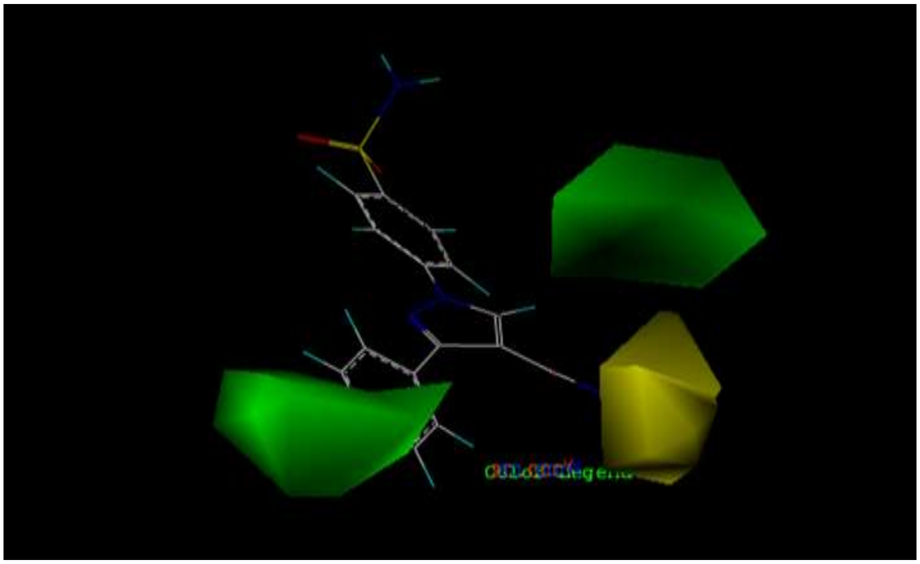

Figure SF6

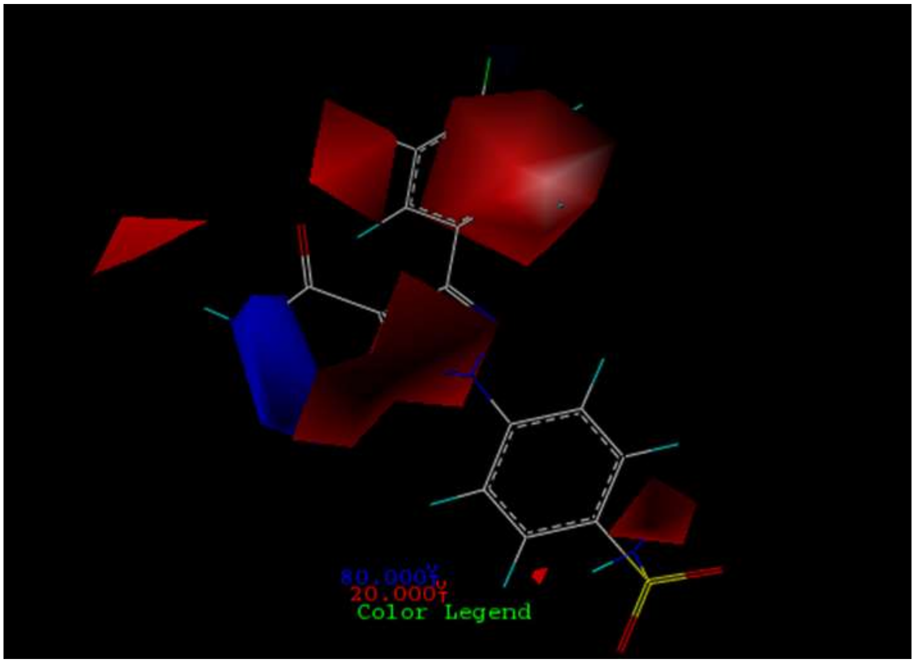

Figure SF7

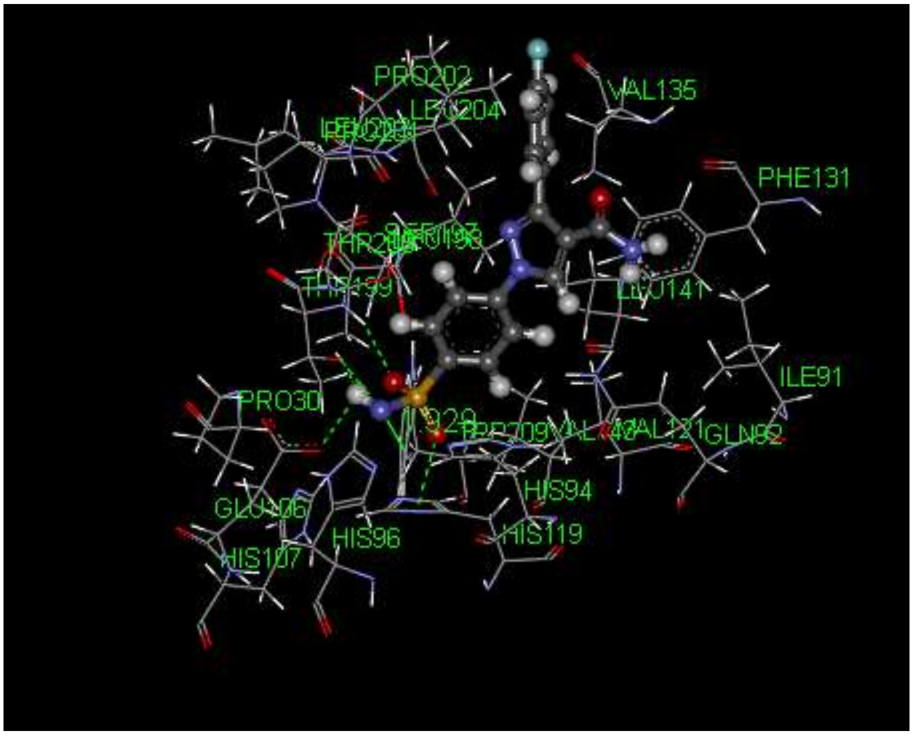

Figure SF8

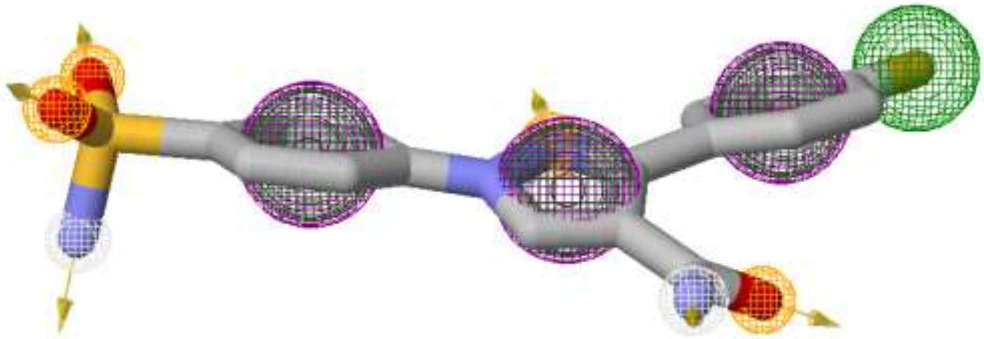

Figure SF9

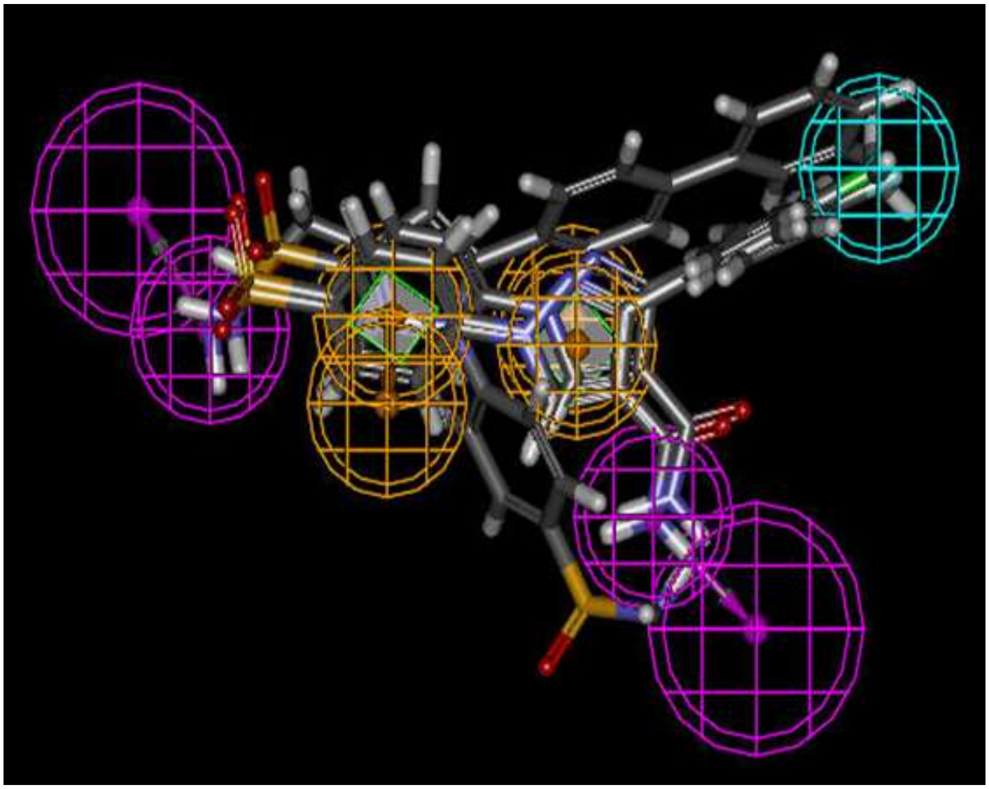

Figure SF10

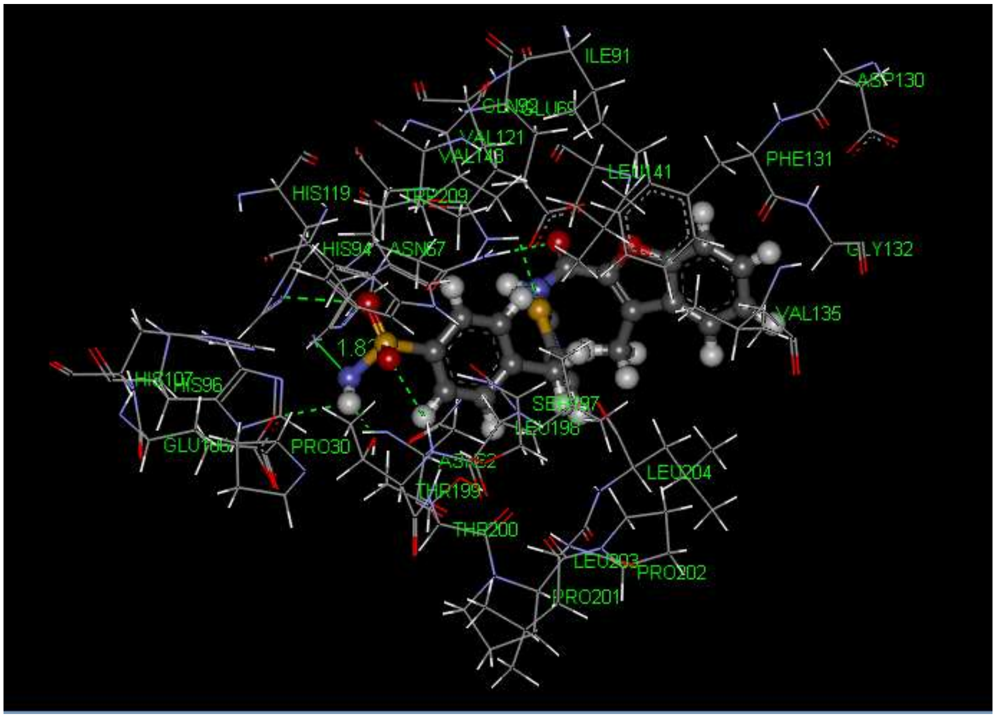

Figure SF11

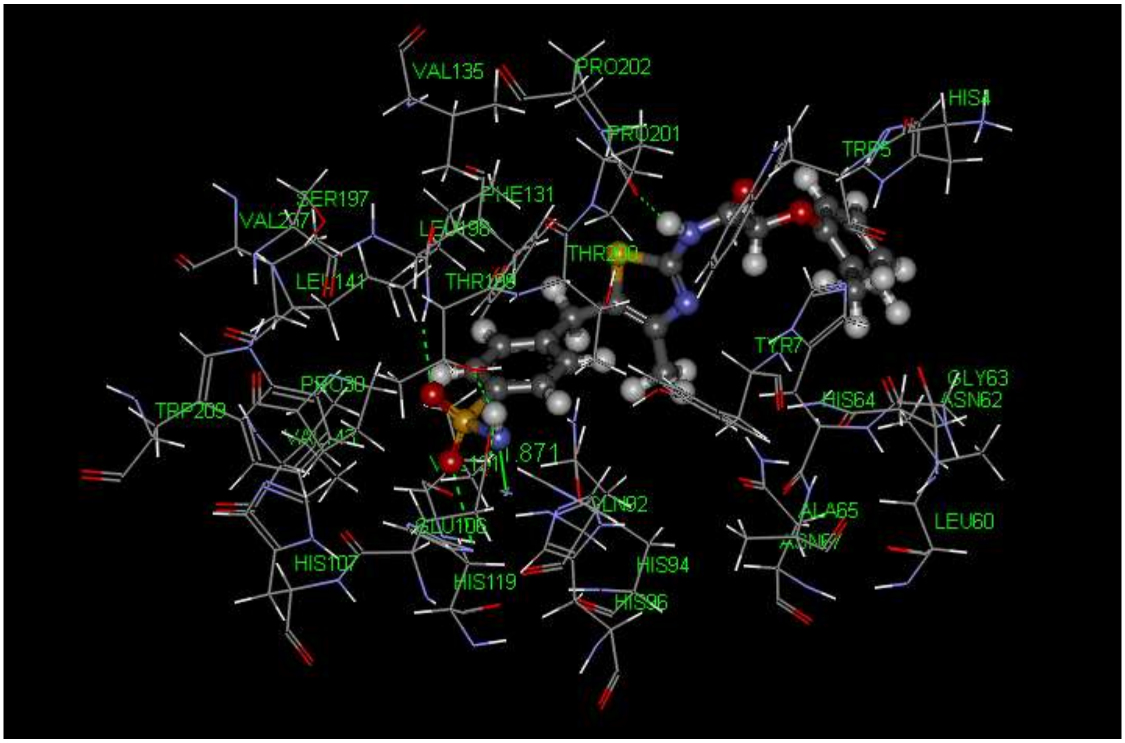

Figure SF12

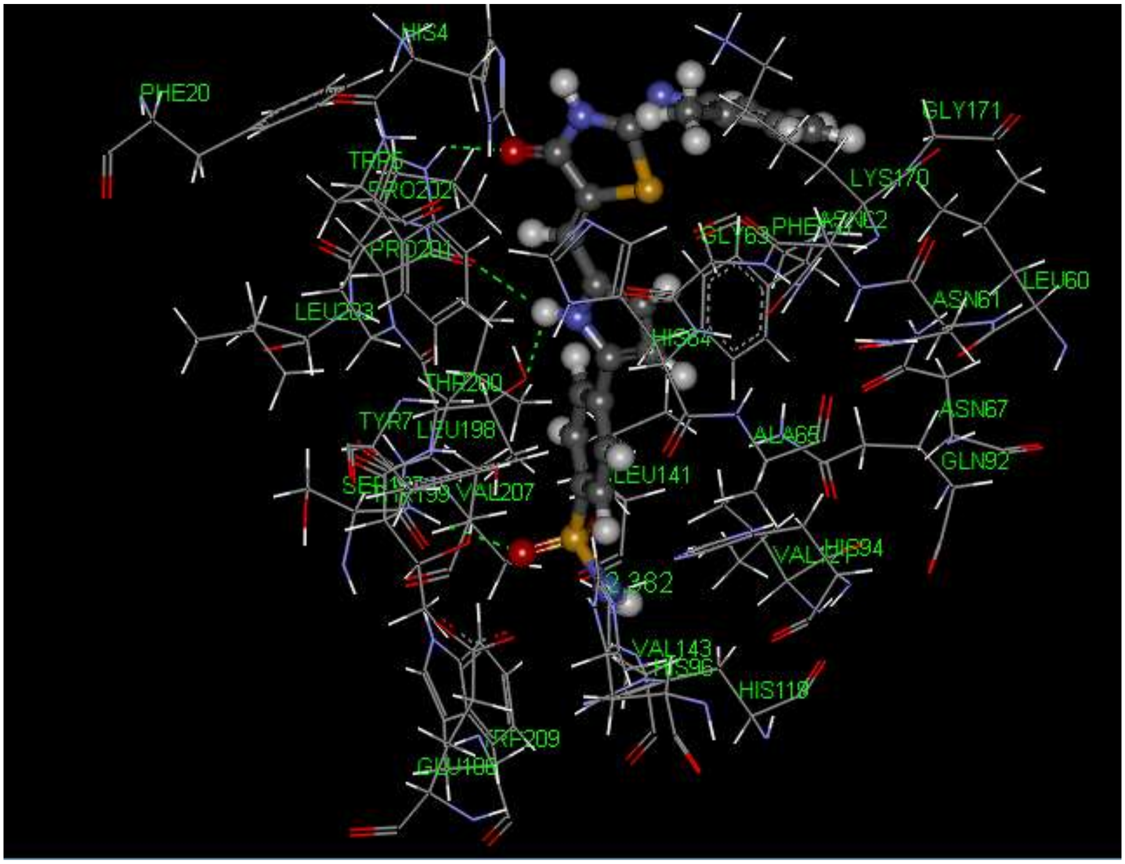

Figure SF13

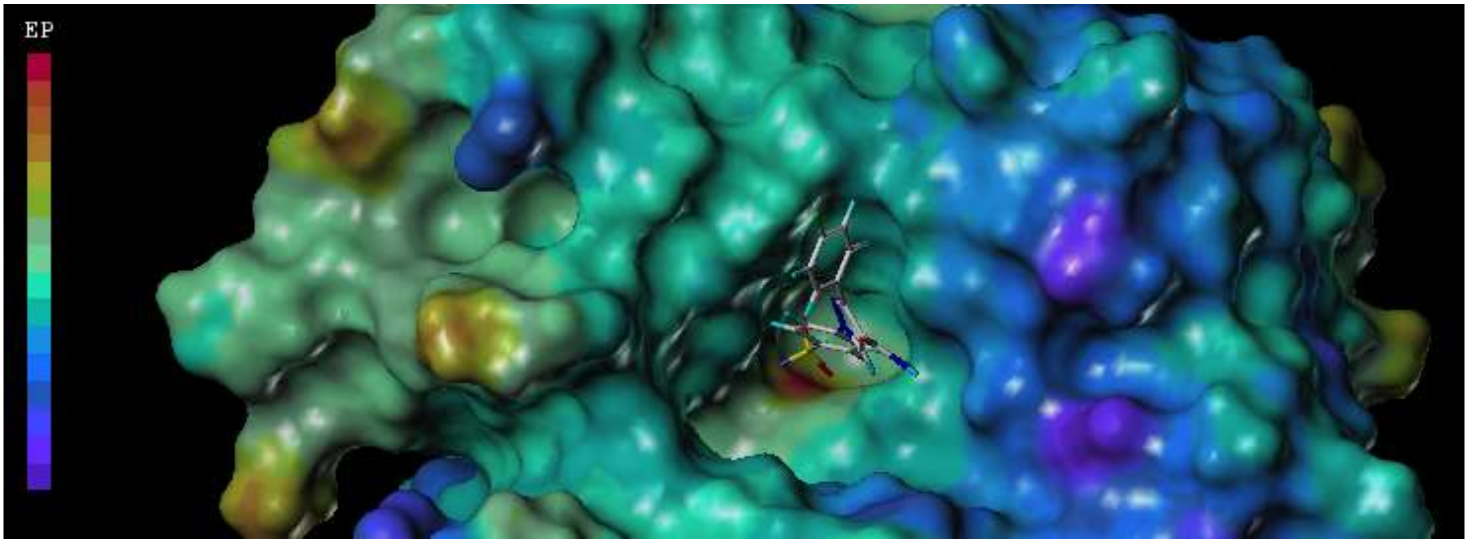

Figure SF14

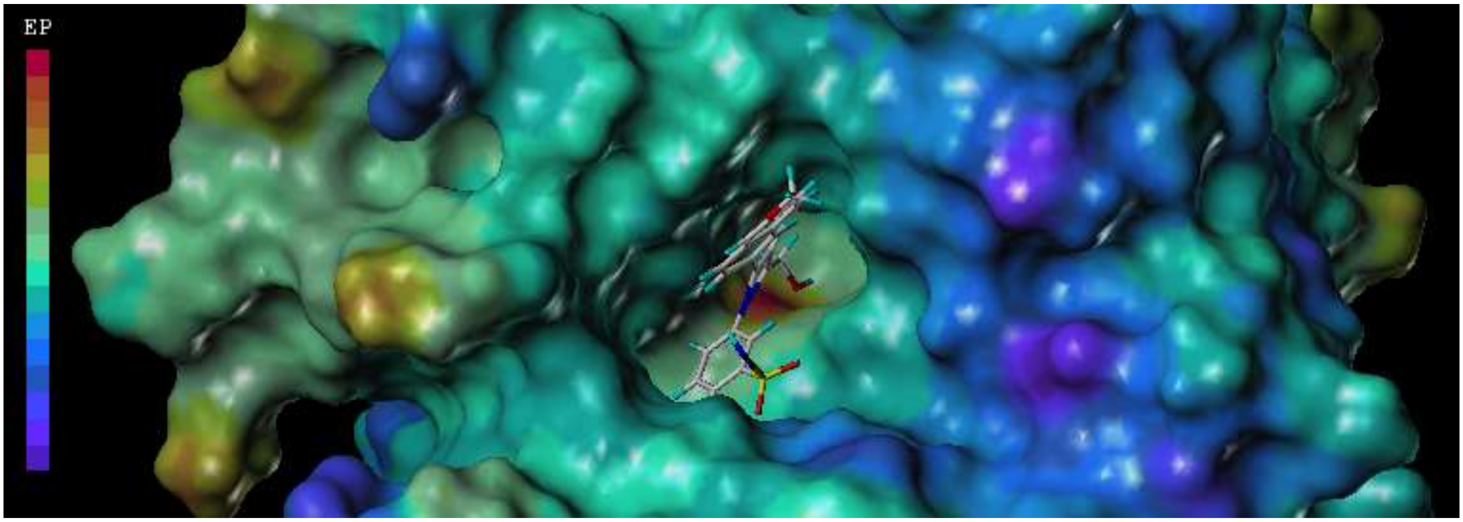

Figure SF15
